# Using Queuing Theory and Simulation Model to Optimize Hospital Pharmacy Performance

**DOI:** 10.5812/ircmj.16807

**Published:** 2014-03-05

**Authors:** Mohammadkarim Bahadori, Seyed Mohsen Mohammadnejhad, Ramin Ravangard, Ehsan Teymourzadeh

**Affiliations:** 1 Health Management Research Center, Baqiyatallah University of Medical Sciences, Tehran, IR Iran; 2 School of Management and Medical Information Sciences, Shiraz University of Medical Sciences, Shiraz, IR Iran; 3 Department of Health Management and Economics, School of Public Health, Tehran University of Medical Sciences, Tehran, IR Iran

**Keywords:** Queuing Theory, Patient Simulation, Pharmacy, Hospitals

## Abstract

**Background:**

Hospital pharmacy is responsible for controlling and monitoring the medication use process and ensures the timely access to safe, effective and economical use of drugs and medicines for patients and hospital staff.

**Objectives:**

This study aimed to optimize the management of studied outpatient pharmacy by developing suitable queuing theory and simulation technique.

**Patients and Methods:**

A descriptive-analytical study conducted in a military hospital in Iran, Tehran in 2013. A sample of 220 patients referred to the outpatient pharmacy of the hospital in two shifts, morning and evening, was selected to collect the necessary data to determine the arrival rate, service rate, and other data needed to calculate the patients flow and queuing network performance variables. After the initial analysis of collected data using the software SPSS 18, the pharmacy queuing network performance indicators were calculated for both shifts. Then, based on collected data and to provide appropriate solutions, the queuing system of current situation for both shifts was modeled and simulated using the software ARENA 12 and 4 scenarios were explored.

**Results:**

Results showed that the queue characteristics of the studied pharmacy during the situation analysis were very undesirable in both morning and evening shifts. The average numbers of patients in the pharmacy were 19.21 and 14.66 in the morning and evening, respectively. The average times spent in the system by clients were 39 minutes in the morning and 35 minutes in the evening. The system utilization in the morning and evening were, respectively, 25% and 21%. The simulation results showed that reducing the staff in the morning from 2 to 1 in the receiving prescriptions stage didn't change the queue performance indicators. Increasing one staff in filling prescription drugs could cause a decrease of 10 persons in the average queue length and 18 minutes and 14 seconds in the average waiting time. On the other hand, simulation results showed that in the evening, decreasing the staff from 2 to 1 in the delivery of prescription drugs, changed the queue performance indicators very little. Increasing a staff to fill prescription drugs could cause a decrease of 5 persons in the average queue length and 8 minutes and 44 seconds in the average waiting time.

**Conclusions:**

The patients' waiting times and the number of patients waiting to receive services in both shifts could be reduced by using multitasking persons and reallocating them to the time-consuming stage of filling prescriptions, using queuing theory and simulation techniques.

## 1. Background

Major changes and challenges in health fields and the important position of outpatient treatment in whole system of health services ascertains the necessity of accurate planning and management of these centers (1-4). Because of the limited equipment and human resources, hospital managers try to provide the best possible services for patients and take some convenient measures to improve satisfaction and profit by optimization of existing situation (5-7). There are various quality assurance indicators. In outpatient hospital departments, the main indicator is patients' waiting time (8-10). To improve service quality, the waiting and service times are considered (11). Lots of people choose private centers for their high-quality services and, accordingly, the overcrowding and long waiting times in public health centers are being prevented (12-14). Long waiting times in health centers can lead to increase the severity of disease and cause socio-economic costs. The results of some studies on assessing patients' satisfaction showed a direct correlation between patients' satisfaction and the waiting times, and indicated a negative effect of long waiting times on total patient's perception of service quality (15-17). A pharmacy, like other service-centered industries, works in a competitive environment (18). Although customers communicate with different departments of hospital, a high percentage of outpatients refer to the pharmacy for their drug needs. These patients come to pharmacy after being visited in medical clinics or other hospital departments, where prescription filling is being performed. Queues are made when the amounts of patient's arrivals exceeds the rate of service delivery. Long waiting times affect the pharmacy's efficiency and cause patients' dissatisfaction (19, 20). The results of some studies indicate a strong correlation between total customer satisfaction and their satisfaction from drug services (21-23). The main variables determining the waiting times for an outpatient pharmacy include: 1: The model of receiving prescriptions; 2: The sequence of work; 3: The percentage of staff working (on duty); and 4: job interactions among providers working in the pharmacy (24). The Queuing theory is an analytical survey of waiting in queues as a comprehensive and scientific background in the operation management (25). Queuing theory uses mathematical models and operational measurements to evaluate and increase customer flow in the whole queuing network (26, 27). Queuing theory has been used widely in some health areas such as planning emergency care centers, transplantation waiting lists and pharmacy affairs (28-33). Queuing theory and its applications have been paid less attention in the pharmacy performance management (27). In the pharmacy, the queuing theory can be used for assessing different variables, like the time of filling prescription, patient's waiting times, the time of drug delivery, consultations, ranking personnel and number of pharmacist or technician required to be employed. It can also be used in pharmacies with huge customer flows. Waiting in a queue is just one part of the queuing system. Queuing network is described by four elements: Entrance, queuing model, mechanism of service delivery and cost structure (27).

Simulation is a reliable tool for evaluating and analyzing the plane of a new system or making required corrections in the current system and also suggests the needed reforms in controlling system and operational roles. Simulation employs a method to present information obtained from a constructed model based on observing work flow rotation in the current situation and other related variables (34-38). Simulation is an approved tool for accurate and evidence-based decision making and provides excellent outcomes in assessing and planning complicated and uncertain systems such as health systems. This technique increases our perception of the main problems and it's possible solutions through modeling and animating health system (39). In recent years, simulation has been used in health systems increasingly due to the progressive and increasing complexity of health systems, considerable capacity of simulation for modeling complex and uncertain systems, and prominent improvement in simulation softwares (40).

## 2. Objectives

This study aimed to investigate the queuing network in a military hospital outpatient pharmacy, to model its queuing system by simulation and, finally, to develop necessary strategies for improving service quality after reviewing proposed scenarios.

## 3. Patients and Methods

This was a descriptive-analytical study conducted in a military hospital in Iran, Tehran in 2013. This hospital was a public, specialized and subspecialized, referral, and teaching hospital with 40 departments including clinical, financial and administrative ones. The studied hospital had 812 available beds, 81% average bed occupancy rate, and average length of stay 4 days. On the other hand, this hospital outpatient pharmacy was a specialty pharmacy, which provided services to the patients in two shifts, morning (from 8 A.M. to 12 A.M.) and evening (from 14 P.M. to 18 P.M.). The number of personnel in service in the morning shift was 4 (2 for receiving, 1 for filling prescriptions and 1 for delivering them) and in the evening shifts was 5 (2 for receiving prescriptions, 1 for filling and 2 for delivering prescriptions). The study population consisted of all patients referred to this hospital outpatient pharmacy in two morning and evening work shifts. The sample size was calculated using the findings of previous study (41) and the following formula, assuming α = 0.05, δ = 5.12 minutes and d = 1 minute per work shift:

*n* = ((1.96 × 5.12) / 1)^2^ = 100.705

Therefore, we should select 200 patients in two shifts and because of eliminating possible sample, 220 patients were selected using systematic random sampling method in which the patients referred to the hospital pharmacy were studied from 8 A.M. to 18 P.M. in standard 30-minute intervals (assessed by a chronometer) and data on arrival times and the times of passing the prescription, filling prescription, referring to cashier, drug delivery and departure were collected by an observer using preplanned forms. Then, the collected data were analyzed using SPSS (Statistical Package for the Social Sciences) software, version 18 (SPSS Inc, Chicago, IL). In order to analyze queuing theory variables using the analyzed data, at first, the arrival rate (λ) which was the number of clients entered to the pharmacy during the standard study time (30-minute intervals), and the rate of receiving services (µ) which was the time period of giving services to each patient per 30 minutes were determined as pharmacy queuing system, and then according to these two parameters, the queuing network performance indicators of the hospital current state of pharmacy in two studied work shifts including the average number of patients receiving services, the average number of patients referred to the pharmacy, the average waiting time spent by patients in the queues, the average time that patients spent in the pharmacy, the average time of not receiving immediate services after entrance to the pharmacy, and the utilization indicator of system were calculated. Then, a simulation model was developed using ARENA software, version 12 (Rockwell Softwares Corporation). After completing the queuing model, four scenarios were explored. In this phase, based on analysis of the queuing theory and pharmacy simulation model outputs, operational strategies for improving the hospital outpatient pharmacy's queuing system through available resources and optimizing the service delivery mechanism were proposed. This study was approved by the Medical Research Ethics Committee of the Baqiyatallah University of Medical Sciences in December 2012 (CH/7018/98).

## 4. Results

In this study, the patients were from an infinite population. The hospital pharmacy’s queuing network was based on FIFO (First-In, First-Out) method in both morning and evening work shifts, and patients who referred earlier would be in upper priority of filling prescriptions. The pharmacy's queuing model in two work shifts was one-lined, multi-server and multi-phase. According to the field observations, 10-15% of patients requested over-the-counter drugs and 85-90% of them requested the prescribed drugs. Initial data analysis showed that the amount of patients' arrival to the pharmacy in the morning and evening shifts followed Poisson distribution, while the rate of providing services followed Exponential distribution (Table 1). The queuing network performance indicators of the pharmacy are shown in Table 2. Using simulation technique, pharmacy's queuing network was modeled according to 3 main stages of service delivery process and subsequent queues made in each related stations in the both two work shifts. The output of simulated model of the morning shift showed that 32.5% of all patients had been referred to the pharmacy between 10 to 11 A.M and patients spent 34 minutes (by average) in the pharmacy. Patients waited, on average, 46 seconds in the delivery station and the queue length was less than one person (0.1) and 90.83% of patients had faced with no queue in this station (Table 3). The average waiting time in the filling prescription stage, before referring to the cashier was 24 minutes and 24 seconds per patient and 61.7% of patients had faced with a queue length of more than 10 persons. Therefore, this stage was the most time-consuming stage of the service delivery process. During delivery stage, the average waiting time was 1 minute and 33 seconds per patient and its queue length was a little longer than that in the receiving prescription stage and was equal to 48%people. The probability of queuing in this stage was 37.5%. The output of the simulation model showed that in the morning shift no queue was made in the receiving prescriptions and drug delivery stages.

**Table 1.** Arrival and Service Rate in Pharmacy’s Work Shifts

Work Shifts	Arrival Rate (λ)	Service Rate (µ)
**Morning**	14.87	14
**Evening**	12.5	11.5

**Table 2.** The Queuing Network Performance Indicators in the Morning and Evening Work Shifts

Variable	Formula	Morning Shift	Evening Shift
**Average number of patients receiving services**	*r = λ / µ*	1.06 person	1.08 person
**Average number of patients referred to the pharmacy**	*L = Lq + r*	19.21 person	14.66 person
**Average waiting time in queues**	*Wq = Lq / λ*	36.6 min	32.59 min
**Average total time spent in the pharmacy**	*W = Wq + 30/μ*	38.74 min	35.19 min
**Average time of not receiving immediate services after entrance to the pharmacy**	*Wa = 30 / (sμ-λ)*	0.72	0.66
**Utilization indicator of system**	*ρ = λ / sμ*	0.25	0.21

**Table 3.** The Queue Length in the Filling Prescription Station in the Morning Shift

Queue Length	Queue Description
**0-5 persons**	25.8 of patients faced with a queue length of 0-5 people.
**5-10 persons**	12.5 of patients faced with a queue length of 5-10 people.
**10-15 persons**	23.3 of patients faced with a queue length of 10-15 people.
**15-20 persons**	30.8 of patients faced with a queue length of 15-20 people.
**20-24 persons**	7.5 of patients faced with a queue length of 20-24 people.

The first scenario explored in the morning shift was to decrease the personnel number in receiving prescriptions station from 2 to 1. The outputs of the simulation model based on this scenario are shown in Table 4. The second scenario was about increasing filling prescription personnel from 1 to 2 so that patients could refer to two prescription fillers. The results of simulation are shown in Table 5. The results of the simulation model of present pharmacy queuing network in the evening shift showed that 32.3% of patients referred to the pharmacy were between 16 to 17 P.M and the patients spent, on average, 29 minutes in the pharmacy. Patients waited, on average, 50 seconds in the delivery station and the queue length had been less than 1 person (15%), and 86.02% of patients had faced with no queue. The average waiting time for filling prescriptions and referring to the cashier was 20 minutes and 2 seconds per patient and 30.1% of patients had faced with a queue length of more than 10 persons. Therefore, this stage was the most time-consuming stage of service delivery process.

**Table 4.** Changes Due to the Scenario of Reducing the Number of Prescription Receivers in the Morning Shift

Title	Before Reducing	After Reducing	Changes
**Average queue length **	Less than 1 person (0.1 person)	Less than 1 person (0.2 person)	Increasing 0.1 person
**Average patients' waiting time **	46 sec	1 minute and 2 seconds	Increasing 16 seconds

**Table 5.** Changes Due to the Scenario of Increasing Prescription Filling Personnel in the Morning Shift

Title	Before Increasing	After Increasing	Changes
**Average queue length **	more than 12 persons	Less than 2 persons	Average decrease of 10 persons
**Average patient wait**ing** time **	24 minutes and 24 seconds	6 minutes and 10 seconds	Decreasing 18 minutes and 14 seconds

During delivery stage, every patient waited, on average, for 1 minute and 26 seconds and the queue length was a little longer than that in the receiving prescription stage, which was equal to 0.52 people. The probability of queuing in this stage was 32.3%, it can be said that in the evening shift, no long and time-consuming queue was made in the receiving prescriptions and drug delivery stages. The first scenario in the evening shift examined the reduction of personnel from 2 to 1. The results of this scenario are shown in Table 6. 

The second scenario in the evening shift was to increase the prescription filling personnel from 1 to 2 (Table 7). Therefore, patients could refer to two prescription fillers. The results of the simulation model showed that this would decrease the average queue length from 5 persons to 2.02. On the other hand, average waiting time for patients would decrease to 11 minutes and 18 seconds, which showed a decrease of 8 minutes and 44 seconds.

**Table 6.** Changes Due to the Scenario of Reducing the Number of Drug Delivery Personnel in the Evening Shift

Title	Before Reducing	After Reducing	Changes
**Average queue length **	Less than 1 persons (0.52)	Less than 1 persons (0.64)	Increase of 0.12
**Average patient wait time **	1 min + 26 sec	1 min + 57 sec	Increase of 31 sec

**Table 7.** Changes Due to the Scenario of Increasing the Prescription Filling Personnel in the Evening Shift

Title	Before Increasing	After Increasing	Changes
**Average queue length **	More than 7 persons	Less than 3 persons (2.02 persons)	Average decrease of 5 persons
**Average patient wait time **	20 min + 2 sec	11 min + 18 sec	Decrease of 8 min + 44 sec

## 5. Discussion

Calculating the arrival amount and service rate in the studied hospital pharmacy indicated that the patients’ arrival amount and service delivery rate followed Poisson and Exponential distributions, respectively. Some international studies have found similar results and are consistent with the current study (42-45). In the present study, patients’ arrival amount exceeded service delivery rate, which is consistent with the results of a study conducted in a specialized hospital in 2012 where the entrance rate was 52 and service delivery rate was 6 patients (45). The longest time spent by referred patients to the studied hospital pharmacy, both in the morning and evening shifts, was in the prescription filling stage, while in a study conducted on the patterns of responding to the patients’ waiting times in an outpatient pharmacy, 50.79% of patients’ time had been spent for paying the bill (19) which is inconsistent with the present study results. The results of another study showed that there was a direct correlation between the time of filling prescription and total patients’ waiting time spent in the pharmacy and there was a negative correlation between the time of filling prescription and service delivery rate, (46) which is inconsistent with the results of current study. Other studies, which have used the theory of queuing and simulation models have identified the process problems and have introduced some improvement solutions. In a study conducted in an outpatient clinic of a public hospital, the main problem was a time lag between admitting patients and the start of examining activities in the examination room (41). This problem was resolved by using a simulation model and redefining the physicians’ attendance schedule. The results of mentioned study are consistent with the results of our study. In the current study, in spite of increased service capacity in the evening shift, compared to the morning shift, after adding one needed extra personnel in the service delivery system, the utilization rate decreased by 4%. In another study (45) the capacity of service delivery reduced after increasing the total number of physicians, so that the system performance indicator with 10 physicians was 86.6% that decreased to 78.8% after increasing their number to 11 physicians. After increasing the total number of physicians to 12, 13 and 14, the system performance indicator decreased to 72.2%, 66.7% and 61.9%, respectively. On the other hand, along with the decrease in the system utilization rate due to the increase in the physician’s number, patients’ waiting time in queues and in the system decreased, so that the waiting time decreased from 3.73 minutes with 10 physicians to 0.112 minutes with 14 physicians. These results are similar to the results of our study. In the current study, the approach of improving pharmacy’s queuing network focused on optimizing that using available resources and changing queue’s characteristics including the queuing system and servers’ arrangements. In other words, a simulation model was used after assessing current situation through queuing theory. In a study of using simulation models in the outpatients’ queues (8), two main methods have been mentioned for changing queue’s characteristics including changing the patient entrance process and changing the service delivery process. Because the change of patient entrance process was impossible in the present study due to the random entrance from an unrestricted population, we changed the service delivery process similar to the above mentioned study. The results of a study in a hospital inpatient pharmacy in Malaysia (47) showed a sharp decrease in patients’ waiting time by adding an extra person for receiving prescriptions at the beginning of the service delivery process, which is consistent with the results of current study. In another study, the results showed that there was a significant decrease in the consultation time and total patients’ waiting time if one physician only provided medico-surgical care and another physician provided only dermatological care (24). On the other hand, results of a study (48) showed that prescription filling interruptions and delays can be reduced by employing and hiring appropriate number of employees in the peak periods of referring to the pharmacy. In another study (49) the results showed that removing one or more physicians in each work shift did not have any negative effects on the functions of the emergency ward, if this ward did not provide any services for the non-emergency patients. the results of the current study showed that reduction in the number of servers in some stages of the service delivery process did not have any negative effects on the pharmacy performances, which are similar to the above mentioned study results. Like our study, some other studies have decreased the studied patients’ waiting time through rearranging the combination of the service providers. The results of a study (50) showed that the patients’ waiting time could be kept in an appropriate range by correction of the number and combination of nurses. By examining different scenarios, it was found that the problem of service delivery system was in the triage area and adding another nurse could decrease the patients’ waiting time. In another study in a local hospital’s outpatient clinic (51) researchers found that determining and assigning appropriate number of residents using a simulation model could reduce total patients’ waiting time. In a study on using queuing theory to increase efficiency and effectiveness of hospital emergency personnel (50), reallocation of human resources according to the queue model, without any increase in staff hours of service, could reduce the number of patients leaving the health center without receiving required services. The result of Su and Shih’s study showed that the time spent in the hospital admission unit could be reduced from 17.24 minutes to 3.15 minutes using simulation models for analyzing workflow and the time related to each process as well as rearrangement of related processes. They concluded that queuing theory was an appropriate tool for determining the correct model of personnel arrangement (52). The results of this study confirmed the results of present study which showed that employing multitasking personnel in the pharmacy could change queuing model and improve patients’ queuing status. Gunal and Pidd in their study (53) after examining two scenarios concluded that employing experienced and multitasking personnel could reduce patients’ waiting time and improve service delivery rates. Also, in another study conducted on applying queuing theory in human resources management in healthcare (54), researchers found that patients’ waiting time would be dramatically reduced if the other wards’ personnel or free experienced personnel were used during the peak periods of referring to the hospital. The results of these studies confirmed the results of present study. the study results of a simulation model and multi-stages patients flow in outpatients clinics (55), after reviewing proposed scenarios, showed that the best queuing model was constructed when one patient entered the clinic per 15 minutes and the service delivery time was considered 12 minutes. The results of this study are inconsistent with the results of present study because of our unrestricted population and the impossibility of establishing a pre-ordered patients’ turning system. By reviewing the current situation of the studied pharmacy in both morning and evening work shifts, it was determined that the queuing model was one-lined, multi-stage (multi-phase) and multi-server, which was corrected by establishing parallel service lines in filling prescriptions stage through changing the number of servers. In a study on decreasing waiting times in a Nigerian hospital (56), the patients’ waiting time reduced to 67% by applying queuing theory and changing pharmacy queuing model from multi-queue and one-server to multi-queue and multi-server model. In another study (57) it was recommended that some measures should be taken simultaneously in order to decrease the total waiting time of patients who needed more than one medical treatment. This result is similar to the results of present study. Also, the researchers of a study on reducing patients’ waiting time using simulation in a neurosurgery department (58) concluded that increasing medical residents from 1 to 3 persons could lead to the reduction of the average patients’ waiting time in the studied department, and in the queues from 40 to 12-13 minutes and from 28.45 to 0.4-1, 5 minutes, respectively. Furthermore, in another study (21) the average patients’ waiting time decreased from 167.0 to 55.1 minutes, a decrease of 67%, using queuing theory. Overall, the results of the above mentioned studies confirmed the results of present study.

This study had a limitation, where the over-the-counter drugs and patients requesting such drugs were not considered in the evaluation of scenarios. In conclusion, the studied pharmacy service delivery system could be improved by reallocating the available personnel and also reviewing and modifying patients' flow, without any additional costs, using queuing theory and simulation techniques. In other words, the patients' waiting times and the number of patients waiting to receive services in both shifts of the studied outpatient pharmacy could be reduced by using the multitasking persons and reallocating them to the time-consuming stage of filling prescriptions, without making any perceptible changes in the queuing characteristics of other service delivery stages in the pharmacy. Therefore, a correct and careful analysis of the queuing characteristics of outpatient pharmacies in the hospitals could be achieved by using queuing theory, and the costs and personnel surplus could be reduced by exploring different scenarios using simulation techniques.
